# Limited Near and Far Transfer Effects of Jungle Memory Working Memory Training on Learning Mathematics in Children with Attentional and Mathematical Difficulties

**DOI:** 10.3389/fpsyg.2016.01384

**Published:** 2016-09-21

**Authors:** Michel Nelwan, Evelyn H. Kroesbergen

**Affiliations:** ^1^Lucertis Kinder- en JeugdpsychiatrieRotterdam, Netherlands; ^2^Department of Special Education, Utrecht UniversityUtrecht, Netherlands

**Keywords:** working memory, training, mathematics, attention deficits, ADHD, dyscalculia

## Abstract

The goal of this randomized controlled trial was to investigate whether Jungle Memory working memory training (JM) affects performance on working memory tasks, performance in mathematics and gains made on a mathematics training (MT) in school aged children between 9–12 years old (*N* = 64) with both difficulties in mathematics, as well as attention and working memory. Children were randomly assigned to three groups and were trained in two periods: (1) JM first, followed by MT, (2) MT first, followed by JM, and (3) a control group that received MT only. Bayesian analyses showed possible short term effects of JM on near transfer measures of verbal working memory, but none on visual working memory. Furthermore, support was found for the hypothesis that children that received JM first, performed better after MT than children who did not follow JM first or did not train with JM at all. However, these effects could be explained at least partly by frequency of training effects, possibly due to motivational issues, and training-specific factors. Furthermore, it remains unclear whether the effects found on improving mathematics were actually mediated by gains in working memory. It is argued that JM might not train the components of working memory involved in mathematics sufficiently. Another possible explanation can be found in the training’s lack of adaptivity, therefore failing to provide the children with tailored instruction and feedback. Finally, it was hypothesized that, since effect sizes are generally small, training effects are bound to a critical period in development.

## Introduction

Studies have shown that persistent difficulties in mathematics develop in approximately 5% of school-aged children. An even larger number of children struggle with math on a day-to-day basis, without meeting the criteria for developmental dyscalculia. The underpinnings for math difficulties are manifold, with both cognitive as well as emotional factors contributing to its manifestation and maintenance over time. Recent literature can be divided in multiple foci of interest. Attentional resources (Marzocchi et al., 2002; Dormal et al., 2014), memory processes (Perna et al., 2015), basic number sense (Piazza, 2010; Brankaer et al., 2014; Vanbinst et al., 2015), as well as math anxiety (i.e., Maloney and Beilock, 2012) hold their own in what seems to be a still unintegrated field of research. Mathematics disabilities are recognized as complex neuro-psychological syndromes related to many distinct neurocognitive constructs (Perna et al., 2015) and underlying brain activation (Arsalidou and Taylor, 2011; Metcalfe et al., 2013; Demir et al., 2014). However, relationships between these constructs remain largely unclear.

A large body of research, however, has been conducted in the field of executive functions and their relationship to mathematics. Predominantly, the role of working memory has been a topic of interest. Following the most influential model proposed by Baddeley and Hitch (1977), elaborated upon by Miyake et al. (2000), working memory can be viewed as a cognitive buffer system that allows the temporary storage and manipulation of incoming information necessary for complex tasks. The model describes two slave systems: a visuo-spatial sketchpad and an auditory-verbal component, called the phonological loop. Both slave systems are supervised by a flexible control system, the Central Executive (CE), that is thought to monitor and update information as well as inhibit incoming irrelevant information.

A slightly different approach to working memory has been advocated by Cowan (1988). In this model, working memory is conceptualized as a memory storage in the center of attention, that is limited in both duration and load, so that its contents can be operated on and be integrated with information in long-term memory. Compared to the model of the Baddeley and Hitch model, emphasis is placed on awareness and both voluntary as well as involuntary attentional processes. In this view, not only processing errors of updating can lead to mathematical difficulties. Problems with activating previously learned information or attentional problems would lead to the same behavioral results. This approach is relevant for the present study, as it incorporates attentional processes.

Regarding the role of working memory in the development of mathematical competency the *updating* component of working memory plays an important part (Lee et al., 2012; Van der Ven et al., 2012; Kolkman et al., 2013a). *Updating* represents the ability to update relevant information in working memory and the concurrent ability to inhibit irrelevant information. The construct is part of the CE component of working memory in the Baddeley and Hitch model. Updating seems to be the process involved in keeping track of intermediate outcomes of mental arithmetic and problem solving (Passolunghi and Pazzaglia, 2004, 2005). Involvement of working memory in mental arithmetic in children might follow a developmental path, with the contribution of verbal working memory increasing with age, while that of visuospatial working memory decreases (Van de Weijer-Bergsma et al., 2015c). Children with less working memory capacity tend to perform worse on general mathematical tasks than typically developing peers (Friso-van den Bos et al., 2013).

Despite the fact that most recent contemporary reviews consider working memory to be a relevant factor in mathematics learning and development, contrasting views are advocated by different authors. Considering different aspects of working memory, some authors have found deficits in visual working memory, but no apparent problems in verbal working memory (McLean and Hitch, 1999). Furthermore, Landerl et al. (2004) describe a group of children with mathematics learning disorder in the absence of difficulties in working memory. In this same vein, Temple and Sherwood (2002) found no relationship between working memory and number sense, or working memory and arithmetic capabilities. Working memory is considered by these authors to be neither a necessary, nor a sufficient factor in explaining mathematical difficulties. They advocate a standpoint in which number sense abilities play the most prominent role in a developmental model of mathematical difficulties. Other authors, however, propose a double deficit model: Number sense and working memory both lead to weaker performance in tasks involving mathematical problem solving. Children that exhibit both number sense as well as working memory difficulties, obtain lowest results (Östergren and Träff, 2013; Kroesbergen and van Dijk, 2015). In addition to contrasting research findings, variation in research design, usage of working memory tasks involving counting and covariance of working memory with other domain-general factors, like processing speed, make interpreting results quite difficult (Cowan and Powell, 2014).

### Relationship between Attention Problems and Mathematics

Both clinical observations as well as scientific research have pointed out a high incidence of comorbidity between attention problems and learning disorders. Estimations currently revolve around 30% (DuPaul and Volpe, 2009). Recent evidence from twin-studies suggests a genetic link between symptoms of ADHD, primarily the inattentive symptoms, and mathematic disabilities (Greven et al., 2014). Inattentive children tend to make more mistakes when calculating than attentive children, quite possibly due to working memory deficits frequently found in groups of children with both ADHD and persistent math disabilities. Both working memory and attention are neurocognitive traits that can be viewed as dimensional constructs, with - at the lower end of the continuum- the deficits common in children with ADHD (e.g., Larsson et al., 2012; Martin et al., 2015) and developmental dyscalculia. Based on the above, it could be concluded that a group of children with mild or moderate problems in attention or working memory, in the presence of normal general intelligence, is struggling with mathematics, but doesn’t meet the formal criteria for ADHD or developmental dyscalculia. This is, however, a highly interesting group that is often overlooked and that is -at least in the Netherlands- not eligible for specialized treatment. Teachers could benefit from knowledge on this subject, as well as methods describing how to remediate difficulties with mathematics. However, scientific knowledge about cognitive underpinnings of learning problems is not frequently transferred to the classroom or remediation programs in school.

### Working Memory Training and Mathematics

One of the possible training programs that could possibly find, and has found, its way into the schools is working memory training. Some research suggests beneficial effects of working memory training on mathematics (Bergman-Nutley and Klingberg, 2014; Söderqvist and Bergman Nutley, 2015), but effect sizes are generally small and studies have their methodological limitations, mostly because of a small sample size. The largest sample size to date can be found in a study by Holmes and Gathercole (2013). These authors used Cogmed Working Memory Training in a classroom setting and found considerable long term effects on both working memory as well as mathematics. There is, however, debate in the literature on transfer of working memory training in general. Meta-analyses carried out recently point out effects of training on both verbal and non-verbal working memory, but authors disagree on the matter of far transfer. Melby-Lervåg and Hulme (2013) conclude that at follow-up limited near transfer effects could be measured and there is no clear evidence of generalization of training to other skills. Specifically, for arithmetic, effect sizes are generally low and non-significant. On the other hand, two recent meta-analyses showed long term effects of working memory training on measures of attention in everyday functioning (Shinaver et al., 2014; Spencer-Smith and Klingberg, 2015), which should be beneficial for learning behavior and for outcomes of academic performance, including mathematics. Possibly, effects of working memory training on mathematics are indirect and depend on instructions and training of mathematical skills after gains in working memory have been made. The present study elaborates existing literature by addressing this issue. The questions raised by existing literature are whether children with attentional problems and problems in working memory (a) profit from training working memory and (b) show more gain in a training of mathematical abilities than their untrained peers.

To address these two questions, we trained working memory in a sample of children in primary schools using Jungle Memory training (JM) for 8 weeks, before training basic arithmetic abilities (addition, subtraction, multiplication, and dividing) using a Dutch computer based adaptive arithmetic training (Math Garden) for 8 weeks and compared this group to two control groups. We measured transfer to both verbal and non-verbal working memory tasks and a speeded arithmetic task. Based on some previous studies, we expected a limited transfer to mathematical abilities directly after working memory training but a larger training effect of the arithmetic training compared to our control groups. Current literature, however, is not clear about this issue and the direction this hypothesis is leading, giving us other concurring hypotheses to consider. Based on the meta-analysis by Melby-Lervåg and Hulme (2013) one could hypothesize that all groups would profit equally from the mathematics training. A third possibility would be that performance in mathematics depends on working memory as a base engine, in which case both groups training working memory would perform equally well and better than the group training mathematics only.

## Materials and Methods

### Participants

Children (*N* = 64) were recruited from regular elementary schools in The Netherlands, primarily in the Rotterdam area. Teachers of different schools were asked to participate and they selected the children based on the inclusion criteria provided to them. Children were eligible to participate if they were 9–12 years old when training started (Grades 4–6), had difficulties in mathematics (scores on standardized school based tests below or far below average), and were observed to have attentional difficulties (above average scores on a standardized rating scale – Scholte and van der Ploeg, 2005) as rated by their teachers. Working memory was evaluated by the teachers as well. They were asked to fill out the BRIEF (Behavior Rating Inventory of Executive Function), a rating scale measuring a variety of executive functions, including working memory (Huizinga and Smidts, 2010). Children were, however, not excluded when they did not exhibit specific working memory problems as rated by their teachers. Descriptive statistics can be found in **Table 1** Children with below average scores on reading or reading comprehension or known psychiatric disorders other than ADHD were excluded from participation.

**Table 1:** Means of the groups on descriptive measures.

Condition	JM+MT (*N* = 21)	MT+JM (*N* = 24)	MT only (*N* = 19)		
			
Measure	*M* (*SD*)	*M* (*SD*)	*M* (*SD*)	*F*	*p*
Sex (M/F)	13/8	13-Nov	15/4		
Age	11.03 (0.96)	10.50 (0.90)	10.86 (1.00)	1.823	0.170
Working memory teacher^∗^	20.31 (6.12)	19.33 (5.68)	20.45 (5.48)	0.776	0.465
Attention teacher^∗∗^	9.5 (7.04)	9.94 (7.07)	9.60 (6.27)	0.036	0.967
Mathematical abilities^∗∗∗^	87.14 (22.92)	83.00 (27.70)	85.11 (25.12)	0.146	0.864
Symbolic number comp.	0.95 (0.05)	0.96 (0.04)	0.96 (0.04)	0.030	0.971
Non-symb. number comp.	0.73 (0.09)	0.71 (0.04)	0.74 (0.06)	0.801	0.454
Number Line	0.87 (0.14)	0.79 (0.22)	0.87 (0.17)	0.092	0.913
Go/NoGo	5.19 (3.17)	5.95 (4.27)	6.10 (3.70)	0.350	0.706

*JMT, Jungle Memory training; MG, Math Garden. ^∗^Behavior Rating Inventory of Executive Function (BRIEF) raw score. ^∗∗^ADHD-questionnaire (AVL) raw score. ^∗∗∗^Arithmetic Tempo Test (TTR) raw total score.*

Parents received written information on the study and we obtained their written consent, in accordance with the Declaration of Helsinki, before starting the assessments and training. The study was approved by the ethics committee of the Faculty of Social and Behavioral science, Utraecht University (FETC14-022).

### Tests

There were three main variables of interest in this study: verbal and spatial working memory and mathematical ability. The tests that were used for measuring working memory were (1) a visuospatial working memory task called ‘Lion Game,’ and (2) a verbal working memory task called ‘Monkey Game’ (Van de Weijer-Bergsma et al., 2015b,c). Tasks were administered on a computer with headphones in the school setting, supervised by a student who was present the whole time.

The Lion Game is a visual-spatial complex span task, in which children have to search for colored lions. Children are presented with a 4×4 matrix containing 16 cells. In each trial, eight lions of different colors (red, blue, green, yellow, and purple) are consecutively presented at different locations for 2000 ms. Children have to remember the last location where a lion of a certain color has appeared and use the mouse to click on that location after the sequence has ended. The tasks consist of five levels each of four items, in which working memory load is manipulated by the number of colors children have to remember and update. No cut-off rules are applied (Van de Weijer-Bergsma et al., 2015b). Proportion correct responses were collected. Reliability and validity of The Lion Game have been studied recently (Van de Weijer-Bergsma et al., 2015b). Good internal consistency reliability (Cronbach’s α = 0.87), satisfactory test–retest reliability (α = 0.71) and good concurrent (α = 0.51) and predictive validity have been found. Scores on The Lion Game appear to be a significant predictor of math ability.

The Monkey Game is a verbal span-backward task, in which children have to remember and recall different words backward. Children hear spoken words (i.e., moon, fish, rose, eye, house, ice, fire, cat, and coat). In Dutch, these words are some of the words first learned in reading by children in first grade. Children have to remember the words and recall them backward, by clicking on the written words presented visually in a 3×3 matrix. The task consists of five levels each of four items, in which working memory load is manipulated by the number of words children have to remember and recall backward, ranging from two words in level 1 to six words in level 5. No cut-off rules were applied (Van de Weijer-Bergsma et al., 2015c). Proportion correct responses were collected. The Monkey Game has good internal consistency (Cronbach’s α ranging from 0.78–0.89) and shows good concurrent and predictive validity (Van de Weijer-Bergsma et al., 2015a).

The test used to measure mathematical abilities was the Arithmetic Tempo Test (Tempo Toets Rekenen, de Vos, 1992), a fast paper-and-pencil screening instrument. Five columns are presented, each with 40 arithmetic exercises: addition, subtraction, multiplication, division, and mixed, slowly increasing in difficulty. All problems consist of two-operant equations with outcomes smaller than 100. Pupils are instructed to solve as many problems as they can within a 1-min limit per column. Test–retest reliability was computed in a study by Van de Weijer-Bergsma et al. (2015c) and ranged from α = 0.84–0.87 after 4 months and from α = 0.82–0.86 after 8 months. Combined number of correct answers in the five columns was used as an outcome measure in this study.

To check whether there were group differences in number sense abilities or inhibition, four tasks were administered during the first assessment only. Children were given (1) a symbolic number task that asked them to evaluate which of two numbers was greatest, (2) a non-symbolic number task on which they were asked to evaluate the number of dots presented on either end of the screen. (3) A number line task was presented on which participants had to place a given number on a line that ranged from 0 to 100. A further description of these tasks, as well as information on reliability and validity can be found in an article by Kolkman et al. (2013b). (4) An inhibition task following a Go–NoGo paradigm (De Weerdt et al., 2013) concluded the assessment. Speed and accuracy were measured in these tasks. These tasks were administered individually. As can be seen in **Table 1**, groups did not differ meaningfully in performance on these tasks.

### Intervention

Jungle Memory^TM^ (2008) is a web-based memory training program aimed at 7–16 year-old children. In comprises of three interactive computer games with up to 30 levels of difficulty in each game to train working memory. Each game trains different aspects of working memory and provides the student with regular feedback of progress, both during training and in the back-end of the program. Game 1 (Quicksand) involves memory for and later use of word endings, Game 2 (Code Breaker) features mental rotation of letters, and Game 3 (River Crossing) involves sequential memory of mathematical solutions. Motivational features in the program included positive verbal feedback, a display of the user’s best scores, percentile rankings, and the number of ‘super monkeys’ collected as a result of successfully completing training levels (Alloway et al., 2013). Completion of all three tasks can be obtained in 20–25 min. The program can be found on a website by Memosyne Ltd (2011) – http://lb.junglememory.com.

The Math Garden (Van der Maas et al., 2009) is an adaptive web-based game in which children are able to train their math skills. It comprises of different games of which five calculation-based games (addition, subtraction, multiplication, division, and speed) were used during this study. Children are presented with 10 math problems per game during which they received direct feedback on their answers. To encourage motivation, children are presented with a virtual garden that grows and blooms depending on the effort and progress. The website for Math Garden can be found on http://www.rekentuin.nl.

### Procedure

**FIGURE 1:**
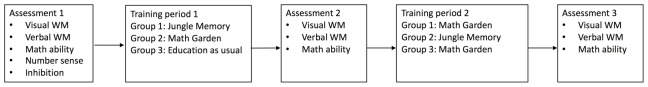
**Assessment and treatment procedures**.

First, children were randomly assigned to three groups. These groups were given three different treatment procedures: (1) the experimental group received Jungle Memory Training (JM) first (8 weeks), before starting mathematics training [MT (mathematics training), 8 weeks]; (2) one control group that received the mathematics training first, before commencing JM, and (3) a second control group that received education as usual in the first period and MT the second period. Assessment occurred three times: prior to training, after 8 weeks and post training. This was done in a group setting on individual computers with headphones. The assessment and treatment procedures are depicted schematically in **Figure 1**

During training periods, progress and effort was monitored by trained undergraduate students visiting schools. In the back-end of both training programs, number of times trained as well as quality of the training can be easily monitored. Undergraduate students visited schools at least once every 2 weeks, giving children feedback on the obtained results, showing their progress and thereby trying to motivate the children. Both children training with JM as well as those training with MT were encouraged to complete four sessions a week by their teachers, as well as the undergraduate students. They were provided training time and a quiet place to train by their teacher during school days. Furthermore, they were provided with training schedules stating the days and the time on which they were supposed to train. Children were given no further rewards or incentives. Both teachers and undergraduate students looked for motivational or technical problems occurring during training. These problems were reported to the corresponding author who attempted to solve the issues. Motivational issues most commonly arose when training or assessment times were planned during a specific activity that was liked by the pupil. These were easily adjusted and no children dropped out. The most common technical issue was a temporary problem with the server of the web-based training programs or the working memory tasks. Sometimes appointments for assessment or training times had to be rescheduled. Children training with JM received feedback and were provided with strategies by the undergraduate students to improve their skills on the training tasks when they failed to pass the task three times in a row. This feedback was based on a protocol that was provided to us by LerendBrein, an organization based in The Netherlands, specializing in training professionals in healthcare and education. Children training with MT received no further feedback; The training program provided all the necessary feedback.

### Data Screening

Missing data showed a number of missing data points, most probably due to server problems during the administration of one the working memory tasks. Missing value counts ranged from 3.1 to 10.1% in one instance, randomly distributed (χ^2^ = 6.011, σ = 1.00). Since the software for the data analysis does not allow for missing data, single imputation based on the series mean was used to replace missing values. The data contained no significant outliers.

### Main Analyses

First, to examine the relationship between progress during JM and gains on the external working memory tasks, Pearson’s *r* correlations were calculated between the working memory pre-test and the reported improvement within the game. Correlations were calculated both for the first and for the second training period, as we were interested in both short and long term effects of JM.

To be able to test the multiple hypothesis formulated without the loss of power, Bayesian evaluation of informative hypotheses was used to examine the relationship between working memory and mathematical abilities. For a detailed introduction to Bayesian statistics, one is directed to Klugkist et al. (2005) and Van de Schoot et al. (2011). This type of analysis is confirmative and provides a quantification of support in the data for each hypothesis discussed and is therefore especially useful in studies with smaller sample sizes. The posterior model probability (PMP) is computed for each hypothesis to quantify the support in the data. If the PMP of one hypothesis is larger than the PMP of the unconstrained hypothesis (*H*_u_), the constraints used to describe the hypotheses are supported by the data. If the PMP of a first hypothesis is larger than the PMP of a second hypothesis, the support in the data is largest for the first. Mark that the sum of the PMPs for a given set of competitive hypothesis is always one.

Next, the Bayes-factor was computed. This is a measure for the degree of support for each hypothesis compared to a hypothesis without constraints. This Bayes-factor was computed by dividing the PMP-value of a hypothesis by the PMP-value of the unconstrained hypothesis, which results in a value showing the evidence in favor of one hypothesis compared with the unconstrained hypothesis.

For effect of JM on gains in working memory we considered two competing hypotheses, for both short and long term effects. All four hypotheses were translated into statistical hypotheses with (in)equality constrained parameters (Klugkist et al., 2005). In this case, the parameters were group means on a verbal and a visual working memory task. For the first training period, the first hypothesis stated that the children that received the JM first would outperform other groups on the working memory tasks, i.e., μ_1_ > μ_2_ = μ_3_ (Model 1). The second hypothesis was that all groups would perform equally well μ_1_ = μ_2_ = μ_3_ (Model 2). For the second training period, the second group -now receiving JM- would have gained more at the working memory measures: μ_2_ > μ_1_ = μ_3._ The alternative hypothesis we formulated was μ_1_ = μ_2_ = μ_3,_ stating that all groups would perform equally well.

For effect of JM on gains made in the MT, three hypotheses were formulated for both the first and second training period. During the first training period, the group receiving MT was thought to perform best, with the group receiving JM performing better than the group receiving no training at all: μ_2_ > μ_1_ > μ_3_ (Model 1). Alternative hypotheses were as follows: μ_2_ > μ_1_ = μ_3_ (Model 2, no direct effect of JM on mathematical abilities), and μ_1_ = μ_2_ = μ_3_ (Model 3, no effect of both trainings). During the second period, the formulated hypotheses were the following: μ_1_ > μ_3_ > μ_2_ (Model 1, stating that the experimental group would outperform the other groups, with the third group -now receiving MT- would gain more than the second group), μ_1_ = μ_3_ > μ_2_ (Model 2, no added effect of JM on MT), and μ_1_ = μ_2_ = μ_3_ (Model 3, suggesting no effects of both MT and JM).

The main question of this study was assessed by examining the total gains on the mathematical abilities outcome measure over both training periods. The first hypothesis stated that children that received the JM first, before the MT, would outperform the groups that either received the JM after the MT or received the MT only, i.e., μ_1_ > μ_2_ > μ_3_ (Model 1). The second hypothesis stated that both groups that trained their working memory would outperform the third group training mathematical abilities only μ_1_ = μ_2_ > μ_3_ (Model 2). The last hypothesis stated that all groups would perform equally well on the last test for mathematical abilities μ_1_ = μ_2_ = μ_3_ (Model 3). All informative hypotheses stated in the section above were compared to the alternative empty hypothesis: μ_1,_ μ_2,_ μ_3_ (Model 0), to protect against incorrectly choosing the hypotheses.

## Results

### Descriptive Statistics

**Table 2** shows the descriptive statistics considering the outcome measures of mathematical abilities, visual, and verbal working memory of our three groups.

**Table 2:** Means and standard deviations on pre-, between and post-tests of mathematical abilities and visual and verbal working memory.

Condition	JM+MT (*N* = 21)	MT+JM (*N* = 24)	MT only (*N* = 19)
	
Measure	*M* (*SD*)	*M* (*SD*)	*M* (*SD*)
Mathematical abilities (*t* = 0)	87 (23)	83 (27)	85 (25)
Mathematical abilities (*t* = 1)	90 (24)	95 (26)	93 (25)
Mathematical abilities (*t* = 2)	97 (24)	95 (26)	95 (28)
Visual WM (*t* = 0)	0.77 (0.09)	0.66 (0.15)	0.73 (0.12)
Visual WM (*t* = 1)	0.74 (0.12)	0.71 (0.18)	0.78 (0.12)
Visual WM (*t* = 2)	0.75 (0.14)	0.70 (0.18)	0.72 (0.18)
Verbal WM (*t* = 0)	0.53 (0.10)	0.54 (0.10)	0.60 (0.09)
Verbal WM (*t* = 1)	0.54 (0.11)	0.52 (0.12)	0.56 (0.11)
Verbal WM (*t* = 2)	0.53 (0.12)	0.52 (0.10)	0.56 (0.10)

**Table 3** shows the descriptive statistics considering the training periods.

**Table 3:** Means and standard deviations of total training intensity of both JM and MT in three groups.

	JM	MT
JM+MT	15.9 (8.3)	21.90 (19.37)


MT+JM	7.55 (5.72)	28.71 (9.00)


MT only	N/A	12.42 (9.11)

### Relationship between Gains Made in JM and Generalization to Non-trained Tasks

**Table 4** shows the Pearson’s *r* correlations between gains obtained in JM by the experimental group, as shown in the stats tab provided by the program, and scores on the non-trained working memory tasks, both after the first (short term) and after the second (long term) period of training. No significant correlations could be obtained. Generalization of the training thus seemed negligible.

**Table 4:** Progression during JM on the three trained tasks in the JM+MG condition and correlations with both short and long term scores on non-trained working memory tasks (visual and verbal WM).

	Visual ST	Verbal ST	Visual LT	Verbal LT
Quicksand	0.272	-0.088	-0.205	0.039
Code breaker	0.176	0.014	-0.207	0.007
River crossing	0.397	-0.016	-0.157	0.167

### Working Memory Training and Gains in Working Memory

**Table 5** depicts the results of Bayesian analysis of the group effects on the working memory outcome measures, BFs (Bayes Factors) and PMPs are presented. It was expected that during the first training period (*t* = 1), the experimental group would gain most on working memory outcome measures, μ_1_ > μ_2_ = μ_3_ (Model 1). Model 2 stated that all groups would perform equally well, μ_1_ = μ_2_ = μ_3_ (Model 2). These were compared to the empty Model 0 (μ_1,_ μ_2,_ μ_3_). For visual working memory, no support was found for our first hypothesis. For verbal working memory, however, the data showed some support for effects of JM. Both the first and the second model received substantial support by our data, but the first model was more likely.

**Table 5:** Bayes Factors (BF) and Posterior Model Probabilities (PMP) of the three models and the visual and verbal working memory outcome measures (*t* = 1 and *t* = 2).

	Model 0		Model 1		Model 2	
	*BF*	*PMP*	*BF*	*PMP*	*BF*	*PMP*
Visual working memory (first period)	1	0.65	0.06	0.04	0.48	0.31
Verbal working memory (first period)	1	0.12	3.99	0.5	3.06	0.38
Visual working memory (second period)	1	0.29	0.83	0.24	1.65	0.48
Verbal working Memory (second period)	1	0.12	3.31	0.39	4.15	0.49

*^∗^First Period: Model 0: μ_1,_ μ_2,_ μ_3_; Model 1: μ_1_ > μ_2_ = μ_3_; Model 2: μ_1_ = μ_2_ = μ_3_.*

*^∗^Second Period: Model 0: μ_1,_ μ_2,_ μ_3_; Model 1: μ_2_ > μ_1_ = μ_3_; Model 2: μ_1_ = μ_2_ = μ_3_.*

During the second period (t2), it was expected that our second group, training with JM, would show more gains in working memory: μ_2_ > μ_1_ = μ_3_ (Model 1), while the alternative hypothesis was that all groups would gain equally well: μ_1_ = μ_2_ = μ_3_ (Model 2). Both for visual and for verbal working memory, most support was found for the second hypothesis, respectively, receiving weak and substantial support by our data.

### Working Memory Training and Mathematical Abilities

**Table 6** shows the results of Bayesian analysis for the mathematics training. During the first training period it was hypothesized that the group training with MT would perform best on the mathematical abilities outcome measure and a smaller training effect could be found for the JM as well (μ_2_ > μ_1_ > μ_3_,Model 1). Alternative hypotheses were formulated: μ_2_ > μ_1_ = μ_3_ (Model 2) and μ_1_ = μ_2_ = μ_3_ (Model 3). Most, but only weak support was found for the second hypothesis, indicating a training effect for MT and not for JM on mathematical abilities.

**Table 6:** Bayes Factors and Posterior Model Probabilities of the four models and gains on mathematics outcome measure (t1–t0 and t2–t1).

	Model 0		Model 1		Model 2		Model 3	
	*BF*	*PMP*	*BF*	*PMP*	*BF*	*PMP*	*BF*	*PMP*
Mathematical abilities (first period)	1	0.36	0.33	0.12	1.4	0.51	0.001	0.00
Mathematical abilities (second period)	1	0.12	3.70	0.44	2.59	0.31	1.12	0.13

*^∗^First Period: Model 0: μ_1,_ μ_2,_ μ_3_; Model 1: μ_1_ > μ_2_ = μ_3_; Model 2: μ_1_ = μ_2_ = μ_3_;Model 3: μ_1_ = μ_2_ = μ_3_.*

*^∗^Second Period: Model 0: μ_1,_ μ_2,_ μ_3_; Model 1: μ_1_ > μ_2_ > μ_3_;Model 2: μ_1_ = μ_3_ > μ_2_; Model 3: μ_1_ = μ_2_ = μ_3_.*

During the second period, the experimental group trained with the MT. It was hypothesized that this group would show most gains in mathematical abilities after this period, followed by the third group, also training with MT: μ_1_ > μ_3_ > μ_2_ (Model 1)_._ Alternative hypotheses were formulated: μ_1_ = μ_3_ > μ_2_ (Model 2) indicating no added effect of JM, and μ_1_ = μ_2_ = μ_3_ (Model 3) suggesting no effect of both JM and MT during the second period. Our data provided most and substantial support for the first model.

**Table 7** shows the results of the analysis on group differences on the mathematical abilities outcome measure, to examine which group benefited most from both trainings, regarding mathematical abilities: BFs and PMPs are presented. Recall that Model 0 represents the alternative (empty) hypothesis (μ_1,_ μ_2,_ μ_3)._ Model 1 stated that the children training with JM first would benefit most from the MT: μ_1_ > μ_2_ > μ_3._ Model 2 stated both groups training with JM would show most improvement on the MT: μ_1_ = μ_2_ > μ_3_, while Model 3 stated that all three groups would perform equally and profit equally from training mathematics. Model 1 received most support from the data, indicating an effect of the working memory training, although the effect seems small and weak compared to the empty Model 0.

**Table 7:** Bayes Factors and Posterior Model Probabilities of the four models and improvement after mathematics training.

	Model 0		Model 1		Model 2		Model 3	
	*BF*	*PMP*	*BF*	*PMP*	*BF*	*PMP*	*BF*	*PMP*
Mathematical abilities	1	0.28	1.35	0.38	0.47	0.13	0.77	0.22

*Model 0: μ_1,_ μ_2,_ μ_3_; Model 1: μ_1_ > μ_2_ > μ_3_; Model 2: μ_*1*_ = μ_2_ > μ_3_; Model 3: μ_1_ = μ_2_ > μ_3_.*

## Discussion

This intervention study compared three groups of children with math difficulties and attentional problems on effects of both a working memory training (Jungle Memory) and a math training (Math Garden). To our knowledge this is the first attempt to combine both training procedures to investigate the effects of working memory training on both measures of working memory and learning mathematics. Prior research has been inconsistent regarding the effects of working memory training and low effect sizes are reported, especially on mathematical abilities, so multiple hypotheses were tested in this study, using Bayesian statistics.

Regarding the immediate and long term effects of working memory training on closely related working memory tasks, mathematical abilities and learning mathematics, results were discouraging. Gains made in JM showed little or no relationship with gains on non-trained working memory tasks. Some support was found for short term gains in verbal working memory only, but long-term retention was not supported by our data. Children generally trained less during the second training period, most likely due to holiday periods and extracurricular activities that occurred during this period or motivational issues. The experimental group, however, trained relatively often, compared to the control groups. It was observed that individual children that trained most, gained most in both verbal working memory and mathematical abilities, but on a group level this could not be confirmed.

Support was found for an effect of MT on gains in speeded mathematics, as was expected. Also, an added effect of JM was found. Children training with JM improved their mathematical abilities directly after training. After completing both JM and MT, the group training with JM first, showed most improvement. The effects, however, are small and were possibly mediated by the amount of training (our experimental group trained more frequently than our control groups that did not train with JM first). Furthermore, it could be argued that JM contains a task in which pupils are required to solve (and retain and update) mathematical problems. Therefore, these children had more practice time, solved more problems, than children that did not train with JM. This fact confounded the results obtained by this study.

Theoretically, working memory is a considerable factor in predicting math problems. However, training working memory with Jungle Memory does not seem to have a profound effect on mathematical abilities, requires supervised practice, and has to be accompanied by specific feedback by a trainer. Several explanations, both theoretical and practical, could be given for these results. It could be argued that Jungle Memory does not specifically train the aspects of working memory relevant to mathematics. Since Jungle Memory does train the updating component of working memory and has a processing speed component, it could be argued that attentional focus and activation of long-term memory, not directly trained by Jungle Memory, would play a crucial role in mathematics. This would give indirect support for the model proposed by Cowan (1988), that emphasized these components of working memory. Another explanation, and one that fits the Baddeley model of working memory, would be that Jungle Memory does not provide the trainee with the adequate components necessary for training working memory efficiently. Regarding this issue, lack of the program’s ability to adapt efficiently to the level of the trainee -as has been proposed by Klingberg et al. (2002, 2005)- could be an explanation for the results obtained by this study.

In studies that found transfer effects of working memory training in mathematical abilities, authors generally describe a small effect. In these studies, speeded arithmetic tasks were used (St Clair-Thompson et al., 2010; Bergman-Nutley and Klingberg, 2014), just like in the present study, but only addition and subtraction problems were administered. It could be argued that other operations of calculation, specifically division, could load on working memory more heavily, because of the fact that most pupils are less familiar with dividing. The effect of training could therefore be somewhat larger than has yet been shown. However, although this study used a composite score of four basic operations, it is not likely that this kind of effect would have been found, given the small and noisy effects that were obtained. A study by Alloway and Alloway (2009), that did administer a division task, has shown effects on mathematical ability, using the same working memory training as in the present study. This study, however, was underpowered and effects on improvement in mathematics were small. Another study (Holmes et al., 2009) only found long term effects (6 months) of WMT on mathematics and used a mathematical reasoning task that measures number sense, in contrast to basic calculating operations. This finding could be similar to the effect that was found in this study, whereas children that trained with JM first, performed slightly better during the second period than their peers. A last study (Kroesbergen et al., 2012) also found effects of working memory training on early numeracy, but was conducted with pupils in kindergarten. This leads to the hypothesis that there might be a sensitive period for working memory training, during earlier development, and that no great effects of working memory training are to be expected when pupils are trained during later classes of primary school.

### Limitations

The present findings are to be viewed in light of several shortcomings. It proved to be very difficult to plan the intervention periods within a school setting. Training periods were interrupted frequently by vacations, sports activities, preparations for end-of-year activities, and other extracurricular enterprises. After the month of May, training compliance dropped both with pupils as well as teachers. Both lack of continuity as well as decreasing training compliance will have influenced our results in several ways. First, training compliance might have caused gains to be less than they could have been. Second, some children expressed their boredom with the tasks that were used during the assessments. This might have led to less effort on the part of the pupils during the last measurement especially.

Another hazard was the fact that pupils were selected from several different schools, which might have had consequences for standardization of child-teacher relationships and teaching material. Different schools use different methods of teaching mathematics, all with slightly different contents and possible effects on outcome measures. The study design doesn’t allow to control for this effect.

The sample size was lower than expected, due to a lower number of eligible children in the different classes, and due to time restrictions. Bayesian analysis was used to control for this issue. Even though the sample size was small, the feedback system provided to us by LerendBrein was quite difficult to carry out, because schools were far apart and only few pupils per school were eligible to participate in this study due to strict inclusion criteria. Feedback provided to the pupils therefore might have been less than optimal during this study. This might have negatively affected both the intensity and efficacy of the training and therefore the obtained results regarding working memory gains and possibly mathematical abilities.

## Conclusion

In sum, this study provides a limited contribution to the literature of working memory training and its near and far transfer effects, by directly examining the effects on mathematics training. On the positive side, computerized mathematics training has a desirable effect on mathematical abilities in children, in higher grades of elementary school, with difficulties in mathematics and attentional problems. Working memory training, specifically Jungle Memory, did seem to have a positive and added effect on outcomes, but it is still unclear if these effects are mediated by improvement of verbal and/or visual working memory. Unfortunately, this could not be confirmed by our study. These findings are in concordance with existing literature. Based on observations during the study, increasing the amount of effort in working memory training might improve the outcomes in working memory and ultimately mathematics. More research would be needed on this matter. The role of working memory and its mechanisms in mathematical abilities remain unclear. Due to its limitations, the results of this study must be considered with caution. Further research has to account for the precise planning of the intervention program, proper support of the children during their training periods and probably a reward system encouraging the children to do their best during measurements. Furthermore, it is recommended that other outcome measures of mathematical abilities are used concurring with the speeded tests used in most studies. More theoretically, training of attention and strategies aiding retrieval from long term memory might be beneficial for children with mathematical difficulties and attentional problems in higher grades of elementary school. Further research is needed to test these hypotheses.

## Author Contributions

All authors listed have made substantial, direct, and intellectual contributions to the work, and approved for publication. EK and MN construed the study together, EK provided the master students that gathered the data. Analyses were carried out by MN while receiving feedback from EK. MN wrote the paper and EK gave constructive feedback an suggestions on improving the paper.

## Conflict of Interest Statement

The authors declare that the research was conducted in the absence of any commercial or financial relationships that could be construed as a potential conflict of interest.
